# Adult nodular lymphocyte‐predominant Hodgkin lymphoma: treatment modality utilization and survival

**DOI:** 10.1002/cam4.1383

**Published:** 2018-02-26

**Authors:** Clayton Alonso, Sunil W. Dutta, Nandita Mitra, Daniel J. Landsburg, Nicholas G. Zaorsky, Surbhi Grover, Jennifer Peterson, Daniel M. Trifiletti

**Affiliations:** ^1^ Department of Radiation Oncology University of Virginia Charlottesville Virginia; ^2^ Department of Biostatistics University of Pennsylvania Philadelphia Pennsylvania; ^3^ Division of Hematology/Oncology Department of Medicine Hospital of the University of Pennsylvania Philadelphia Pennsylvania; ^4^ Department of Radiation Oncology Pennsylvania State University State Park Pennsylvania; ^5^ Department of Radiation Oncology Perelman School of Medicine University of Pennsylvania Philadelphia Pennsylvania; ^6^ Department of Radiation Oncology Mayo Clinic Jacksonville Florida

**Keywords:** Chemotherapy, NLPHL, radiation, survival

## Abstract

Early‐stage nodular lymphocyte‐predominant Hodgkin lymphoma (NLPHL) is associated with a favorable prognosis. Our aim was to evaluate the patterns of care of radiotherapy utilization in this disease and to define the relationship between treatment modality and survival. The National Cancer Database was queried for patients with stages I‐II NLPHL diagnosed from 2004 to 2012. Patients were compared based on primary therapy into four categories: radiotherapy, chemotherapy, both, or neither. Covariate‐adjusted and propensity score‐weighted (PS) Cox proportional hazards models were used, adjusting for potential factors confounding survival. After exclusions, 1420 patients were evaluated, 571 (40%) received radiotherapy alone, 318 (22%) received chemotherapy alone, 351 (25%) received both, and 180 (13%) received neither. Younger patient age (*P *=* *0.001), female gender (*P *=* *0.019), and chemotherapy use (*P *<* *0.001) were associated with decreased radiotherapy utilization. On PS, radiation alone (HR = 0.298, *P *<* *0.001) and chemoradiotherapy (HR = 0.258, *P *<* *0.001) were associated with improved survival compared to no upfront therapy, but the use of chemotherapy alone did not statistically differ compared to no initial therapy (HR = 0.784, *P *=* *0.078). In this large database analysis, over one‐third of patients with early‐stage NLPHL did not receive radiotherapy as a component of initial therapy. The omission of upfront radiotherapy was associated with inferior survival.

## Introduction

Nodular lymphocyte‐predominant Hodgkin lymphoma (NLPHL) represents approximately 5% of all Hodgkin lymphoma and is recognized to have a more indolent course and potentially an improved prognosis when compared with classical Hodgkin lymphoma (cHL) 1. While advanced‐stage (stages III‐IV) NLPHL is treated with upfront chemotherapy, the current standard of care for (stages I‐II) NLPHL in the United States includes involved site radiation therapy, as the preferred or standard option, for all stage I and stage II patients 2. This standard of care for NLPHL is primarily based on retrospective analyses and subgroup analyses of prospective trials 2, 3, 4, 5, 6, 7, 8. In the case of early‐stage cHL, there has been a decline in the United States (US) in radiotherapy utilization that has been associated, in some studies, with inferior survival following diagnosis^9^. This trend is largely related to large, prospective randomized trials supporting a chemotherapy alone treatment strategy, but these trials do not exist in the setting of NLPHL 2.

Previous retrospective series have shown similar outcomes in patients with early‐stage NLPHL treated with radiation therapy with or without chemotherapy with complete response rates of ≥98% and 10‐year recurrence‐free survival of 68–77% 5, 8. More recently, a Surveillance, Epidemiology, and End Results (SEER)‐based analysis has shown similar results, with a 10‐year disease‐specific survival of 93% 1. The same study showed a decrease in radiation therapy utilization from 1988 to 2010, despite an overall survival benefit associated with the use of radiation therapy on multivariable analysis (hazard ratio, HR = 0.64, *P *=* *0.03) 1. Of note, this study was limited in that it was unable to evaluate the utilization and impact of systemic therapy in this patient population, a topic of considerable interest since the advent of the anti‐CD20 antibody rituximab (initially FDA approved in 1997 for relapsed or refractory low‐grade CD20‐positive non‐Hodgkin's lymphoma), which has demonstrated efficacy as monotherapy in the treatment of NLPHL 1, 10, 11, 12, 13.

Our aim was to update and further evaluate the patterns of care of radiotherapy utilization in the treatment of NLPHL in the United States and to define the relationship between treatment modality (i.e., systematic therapy and radiation therapy) and overall survival (OS) following diagnosis.

## Methods and Materials

### Data source and cohort selection

The National Cancer Database (NCDB) is a prospectively collected database led by the American College of Surgeons that collects patient‐level data on patients with cancer diagnoses from participating institutions across the United States and Puerto Rico 14. It is a joint project of the American Cancer Society and the Commission on Cancer of the American College of Surgeons, who execute a Business Associate Agreement that includes a data use agreement with each of its Commission on Cancer‐accredited hospitals. Established in 1989, the database is a nationwide facility‐based comprehensive clinical surveillance resource oncology dataset that accounts for roughly 70% of new cancer diagnoses in the United States annually 14. This study was approved by our institutional review board prior to initiation.

**Figure 1:**
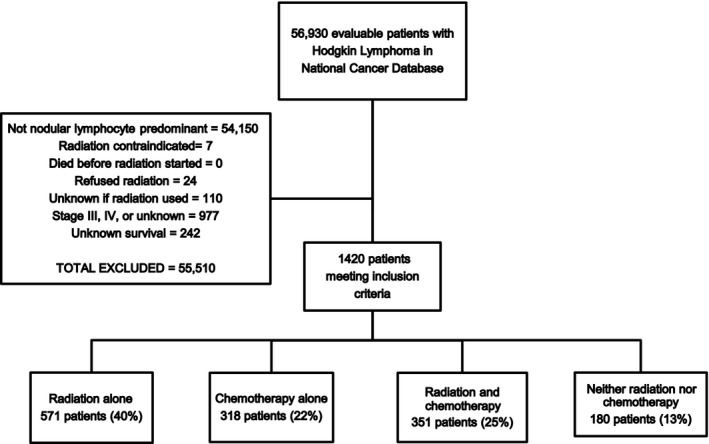
Cohort selection diagram.

The NCDB was queried for patients with known stage I or II NLPHL diagnosed from 2004 to 2012. Figure 1 depicts the cohort selection process. Patients with contraindications to radiotherapy, patients who refused radiotherapy, and patients with unknown radiotherapy utilization were excluded, as were patients with unknown OS following diagnosis (lost to follow‐up). Patient age, year of diagnosis, sex, race, median income of patient ZIP code, distance from patient home to hospital, Charlson/Deyo comorbidity score, insurance status, stage, and the presence/absence of B‐symptoms were extracted from the NCDB dataset for analysis 15. For interpretation and analysis, patients were then categorized into one of four therapy groups based on treatment modality: radiation alone, chemotherapy alone, chemoradiotherapy, and neither.

### Statistical analyses

Chi‐squared tests of independence were performed to compare each categorical demographic and clinical covariate between the four therapy groups. The primary outcome measure of this study was overall survival (OS) following diagnosis of NLPHL based on treatment strategy. Time to death or last follow‐up was evaluated using the log‐rank test as well as an unadjusted and multivariable Cox proportional hazards models including propensity score (PS) inverse probability of treatment weighting (IPTW). All statistical analyses were performed using R version 3.1 (The R Foundation for Statistical Computing, Auckland, New Zealand). The “twang” package in R was used to estimate the propensity scores for each treatment group. We further confirmed that each patient has a nonzero probability of receiving each treatment and that all the covariates were balanced across the four therapy arms. As missing data were infrequent in the included covariates (<5% within each therapy group), indicators for missingness were used in the models. This approach has been shown to be appropriate when using propensity score adjustment 16, 17. Results were considered statistically significant with *P *<* *0.05.

## Results

### Clinical characteristics

After planned exclusions, 1420 patients with early‐stage NLPHL with known therapy and survival information were identified from the NCDB database (Fig. 1). As demonstrated, 571 (40%) patients received radiation therapy alone, 318 (22%) received chemotherapy alone, 351 (25%) received both, and 180 (13%) received neither chemotherapy nor radiation therapy as part of their initial treatment course.

Table 1 describes the patient characteristics of the patient cohort grouped by therapy, with a chi‐squared test to evaluate the difference across groups. The median age among patients was 45 years among patients treated with radiation therapy and 48 years among those in whom radiation therapy was omitted. Both groups were male predominant. Most patients identified themselves as white (69%) or black (22%) in both groups. Of evaluated patients, 58% of identified patients were stage I, the remainder stage II (42%), and clinical stage at presentation predicted for the use of a chemotherapy‐containing approach (*P *<* *0.001).

**Table 1:** Clinical characteristics of the 1420 patients with early‐stage nodular lymphocyte‐predominant Hodgkin lymphoma in the National Cancer Database 2004–2012

Test	No treatment		Radiation only		Chemo only		Both		Chi‐square
	*n*	%	*n*	%	*n*	%	*n*	%	*P*‐value
Total number	180	13	571	40	318	22	351	25	
Age									
≤60	133	74	448	78	239	75	308	88	<0.001
>60	47	26	123	22	79	25	43	12	
Year of Diagnosis (median)									
2004	14	8	35	6	23	7	38	11	0.052
2005	18	10	34	6	26	8	43	12	
2006	10	6	37	6	23	7	32	9	
2007	17	9	46	8	28	9	38	11	
2008	20	11	76	13	35	11	36	10	
2009	22	12	72	13	42	13	41	12	
2010	24	13	76	13	43	14	46	13	
2011	31	17	95	17	49	15	38	11	
2012	24	13	100	18	49	15	39	11	
Sex									
Male	99	55	372	65	201	63	250	71	0.002
Female	81	45	199	35	117	37	101	29	
Race									
White	102	57	385	67	227	71	259	74	<0.001^a^
Black	59	33	122	21	70	22	65	19	
American Indian	0	0	3	1	0	0	2	1	
Asian/Pacific Islander	0	0	11	2	5	2	2	1	
Hispanic	16	9	43	8	12	4	21	6	
Unknown	0	0	7	1	4	1	2	1	
Median income of zip^b^									
<$38,000	49	27	94	16	51	16	51	15	0.021
$38,000–$47,999	27	15	125	22	64	20	85	24	
$48,000–$62,999	42	23	151	26	84	26	98	28	
$63,000+	58	32	196	34	111	35	108	31	
Unknown	4	2	6	1	8	3	9	3	
Distance to Hospital^b^				0					
<25 min	146	81	469	82	259	81	267	76	0.269
25–100 min	25	14	75	13	38	12	64	18	
>100 min	5	3	21	4	14	4	12	3	
Unknown	4	2	6	1	7	2	8	2	
Charlson/Deyo Score									
0	152	84	503	88	275	86	322	92	0.117
1	25	14	55	10	35	11	21	6	
2	3	2	13	2	8	3	8	2	
Insurance									
No	7	4	25	4	16	5	17	5	0.929
Yes	173	96	546	96	302	95	334	95	
Stage									
Stage I	130	72	412	72	126	40	161	46	<0.001
Stage II	50	28	159	28	192	60	190	54	
B‐symptoms^b^									
No	73	41	418	73	143	45	204	58	<0.001
Yes	6	3	14	2	36	11	32	9	
Unknown	101	56	139	24	139	44	115	33	

a Compares only White, Black, and Hispanic groups because of small samples of other subgroups.

b For variables with a significant amount of unknown data points (income, distance, and B‐symptoms), the unknown category was removed in the comparison.

### Factors associated with radiation therapy

**Figure 2:**
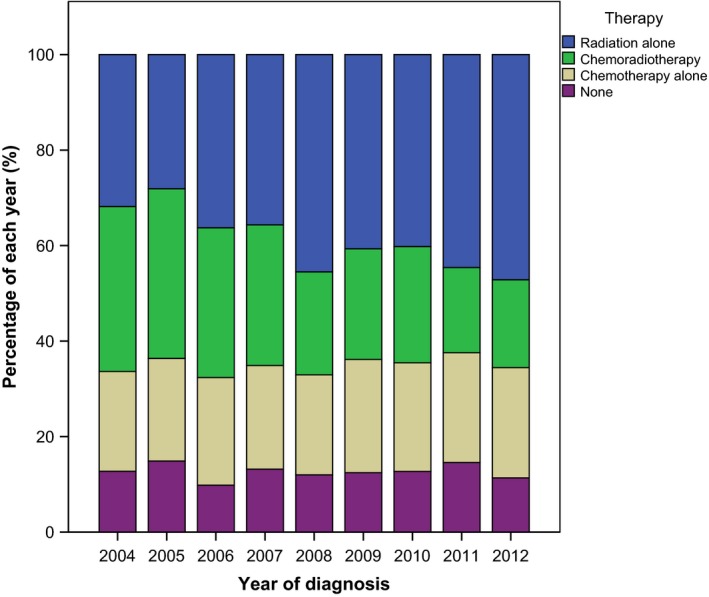
Utilization of therapies over time for patients with early‐stage nodular lymphocyte‐predominant Hodgkin lymphoma in the National Cancer Database (2004–2012).

Table 2 depicts the analysis of factors associated with receipt of radiation therapy. Because of the large percentage of patients with unknown B‐symptoms, this was not included in the utilization model. As demonstrated, factors associated with decreased odds of receiving radiotherapy included younger age (*P *=* *0.001), female sex (*P *=* *0.019), and the use of chemotherapy (*P *<* *0.001) on multivariable analysis. While median household income and Charlson/Deyo score appear to be associated with radiotherapy utilization, a clear trend did not emerge. Of note, stage II NLPHL was not associated with a change in radiotherapy utilization compared to stage I (*P *=* *0.170). Figure 2 demonstrates the relatively stable trend in therapy utilization over the study period (*P *=* *0.640).

**Table 2:** Analysis of factors associated with the receipt of radiotherapy for early‐stage nodular lymphocyte‐predominant Hodgkin lymphoma in the National Cancer Database 2004–2012

	Univariable	Multivariable		
	*P*‐value	OR	95% CI	*P*‐value
Patient age^a^	<0.001	0.987	0.980–0.995	0.001
Year of diagnosis^a^	0.640			
Sex	0.007			0.019
Male		ref		
Female		0.745	0.583–0.952	
Race	0.219			
Median income of zip	0.069			0.022
<$38,000		ref		
$38,000–$47,999		1.764	1.216–2.557	0.003
$48,000–$62,999		1.487	1.048–2.110	0.026
$63,000+		1.313	0.941–1.832	0.109
Distance to Hospital	0.505			
Charlson/Deyo Score	0.069			0.170
0		ref		
1		0.703	0.479–1.030	0.071
2		1.168	0.535–2.551	0.696
Insurance	0.957			
No				
Yes				
Stage	<0.001			0.170
Stage I		ref		
Stage II		0.844	0.663–1.075	
Chemotherapy	<0.001			<0.001
No		ref		
Yes		0.325	0.254–0.415	

a Analyzed as a continuous variable.

OR, odds ratio of receiving radiotherapy; CI, confidence interval.

### Overall survival analyses

**Figure 3:**
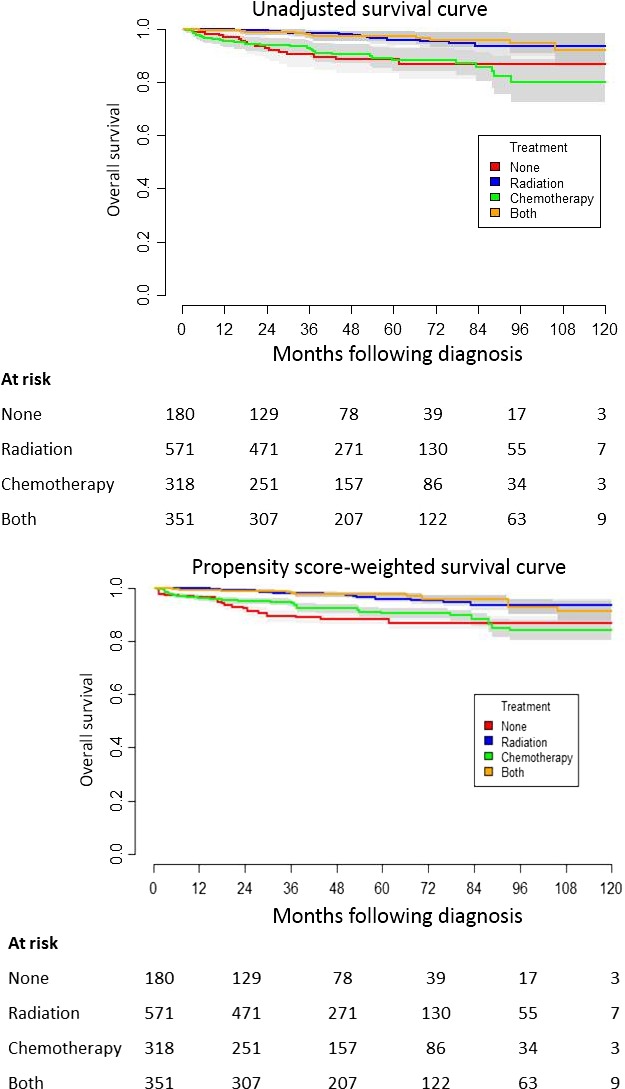
Overall survival following diagnosis among 1420 patients with early‐stage nodular lymphocyte‐predominant Hodgkin lymphoma in the National Cancer Database (2004–2012, shading represents 95% confidence interval).

Median follow‐up among the entire cohort was 48.3 months following diagnosis. Figure 3 provides the unadjusted Kaplan–Meier product limit estimates and PS‐weighted survival curves of OS following diagnosis based on therapy received (*P *<* *0.001). The 10‐year unadjusted OS estimate for the no therapy, radiotherapy, chemotherapy, and chemoradiotherapy groups was 87%, 93%, 80%, and 92%, respectively. Table 3 provides the detailed analysis of factors associated with survival among the study cohort. As demonstrated, older age (HR = 4.082 for age >60 years, *P *<* *0.001), Charlson/Deyo score, and omission of initial therapy were associated with shortened time to death on multivariable analysis.

**Table 3:** Analysis of factors associated with time to death among patients with early‐stage nodular‐lymphocyte predominant Hodgkin lymphoma in the National Cancer Database 2004–2012

	Unadjusted Cox model			Multivariable Cox model			Propensity Score weighted		
	HR	95% CI	*P*‐value	HR	95% CI	*P*‐value	HR	95% CI	*P*‐value
Therapy									
None	1.000	–	–	1.000	–	–	1.000	–	–
Radiation	0.302	0.156–0.586	<0.001	0.294	0.148–0.583	<0.001	0.298	0.211–0.423	<0.001
Chemotherapy	1.023	0.571–1.833	0.939	0.849	0.456–1.582	0.607	0.784	0.598–1.028	0.078
Both	0.271	0.129–0.568	<0.001	0.287	0.131–0.627	0.002	0.258	0.179–0.373	<0.001
Patient age									
≤60				1.000	–	–			
>60				4.082	2.521–6.61	<0.001			
Year of Diagnosis									
2004				1.000	–	–			
2005				1.551	0.647–3.722	0.325			
2006				1.253	0.467–3.36	0.654			
2007				0.391	0.103–1.486	0.168			
2008				1.424	0.548–3.699	0.468			
2009				1.283	0.503–3.278	0.602			
2010				1.596	0.590–4.318	0.357			
2011				1.547	0.541–4.424	0.415			
2012				1.143	0.346–3.774	0.827			
Sex									
Male				1.000	–	–			
Female				0.635	0.393–1.028	0.065			
Race									
White				1.000	–	–			
Black				1.254	0.703–2.238	0.443			
American Indian				7.464	0.937–59.436	0.058			
Asian/Pacific Islander				0.000	NA	0.996			
Unknown				0.750	0.214–2.63	0.653			
Hispanic				0.000	NA	0.997			
Median income of zip									
<$38,000				1.000	–	–			
$38,000–$47,999				0.000	NA	0.999			
$48,000–$62,999				1.265	0.631–2.538	0.508			
$63,000+				0.714	0.339–1.505	0.376			
Unknown				0.601	0.290–1.243	0.170			
Distance to Hospital									
<25 min				1.000	–	–			
25–100 min				1.001	0.515–1.947	0.997			
>100 min				0.898	0.210–3.832	0.884			
Unknown				NA	NA	0.999			
Charlson/Deyo Score									
0				1.000	–	–			
1				1.760	0.971–3.189	0.062			
2				3.508	1.417–8.682	0.007			
Insurance									
No				1.000	–	–			
Yes				0.809	0.235–2.779	0.736			
Stage									
Stage I				1.000	–	–			
Stage II				1.539	0.955–2.479	0.077			

HR, hazard ratio of death; CI, confidence interval; mi, miles.

In comparing outcomes based on treatment modality on multivariable PS‐weighted analysis, radiation therapy alone and chemoradiotherapy were associated with improved survival as compared to no treatment (HR = 0.298 and 0.258, respectively, both *P *<* *0.001). In contrast, the use of chemotherapy alone suggested, but failed to confirm, an improvement in OS compared to no treatment (HR = 0.784, *P *=* *0.078). Additional, similar analyses confirmed these findings through independent groupings of therapy (radiotherapy vs. no radiotherapy, radiotherapy vs. chemotherapy, and radiotherapy vs. chemoradiotherapy, and chemotherapy alone vs. chemoradiotherapy). These results are provided in the supplementary materials (Figs [Link], [Link], [Link], [Link] and Tables S1–S3).

## Discussion

In the largest series on NLPHL to date, our findings are consistent with prior studies which have shown an excellent OS after treatment of NLPHL, with 10‐year OS between 90% and 100% 1, 5, 8. Our findings are also consistent with prior database studies showing an improvement in OS associated with the receipt of radiation therapy 1, 18. In a similar recent study, Odei et al. utilized the NCDB to evaluate factors associated with survival among patients will all stages of NLPHL 18. Their results independently affirm our findings, favoring radiotherapy utilization, in patients with early stage, and also in patients with advanced‐stage NLPHL 18. Given the improvement in OS associated with radiation therapy and chemoradiation seen in these studies, and not in nonradiotherapy containing approaches, there is support of the current National Comprehensive Cancer Network (NCCN) Guidelines recommendation for the use of radiation therapy, possibly in conjunction with chemotherapy, for patients with early‐stage NLPHL 2.

Per the NCCN guidelines, either involved site radiation therapy (preferred) or careful observation (in highly selected patients) is appropriate treatment options for stages IA and IIA NLPHL with combined modality therapy (chemoradiotherapy) for patients with early‐stage bulky disease and patients with B‐symptoms 2. The NCCN does not support the use of chemotherapy alone in patients with early‐stage NLPHL. Despite this consensus recommendation, 22% of patients in our study were treated with chemotherapy alone.

The cause of these variations in practice patterns is complex 9, but we suspect are related to referral patterns to medical oncologists but not radiation oncologists, lack of recognition of the benefit of radiotherapy in the treatment of NLPHL, and the practice of treating NLPHL patients in the same manner as cHL patients. The lowered utilization of radiotherapy among younger, female patients suggests that the possible late effects of radiotherapy (including cardiovascular disease and secondary malignancies such as breast cancer) are a driver in decision‐making in favor of systemic therapy alone approaches. While it does appear that the recent decline in radiotherapy utilization for NLPHL patients identified in previous studies has stabilized 1, our analysis shows that more than 25% of patients receiving treatment for early‐stage NLPHL did not receive radiation therapy.

In the era of rituximab, multiple series have demonstrated that focal radiotherapy is associated with improved disease control compared to systemic therapy 12, 19. Perhaps related to sample size or uncontrolled confounders, these studies failed to demonstrate a difference in OS between upfront systemic therapy and radiotherapy. In contrast, in the current series, we found that patients treated with chemotherapy alone or with observation had inferior OS compared to those treated with radiotherapy (Fig. 3). An important caveat is that during the study period (2004–2012), the NCDB did not code for cytotoxic chemotherapy and targeted therapies differently. Beginning in 2013, rituximab is now to be classified as an immunotherapeutic agent, not a chemotherapy 15. As a result, we suspect that the chemotherapy utilized in the patients in this series consists of those treated with both cytotoxic agents and targeted agents.

The primary limitation of our study is the potential for confounding in a retrospective analysis. It is possible that some uncontrolled variable (B‐symptoms, bulky disease, and interim metabolic imaging such as PET‐CT scans, radiotherapy technique, dose, fractionation, among others) could influence both treatment utilization and survival in this cohort, including treatment bias. However, using propensity score weighting, we adjusted for all confounders that we had available and think have an important effect on treatment decisions and survival. If healthier patients were treated with radiation therapy in ways which were not controlled for in this study, we would overestimate the benefit of radiation therapy.

Of note, the NCDB records the first round of treatment at diagnosis. Therefore, the salvage strategies among this cohort are unknown. However, the OS difference demonstrated here likely includes patients treated with salvage radiotherapy as well as salvage stem cell transplant, raising concern over the potential efficacy of such a salvage strategy on patient survival. Similarly, while OS is reported herein, other critical considerations such as relapse‐free survival, treatment‐induced toxicity, and cause of death are not reported in the NCDB and will be of value in future studies.

This large database analysis shows an improvement in OS associated with the use of radiation therapy in early‐stage NLPHL. Despite this benefit, 35% of patients with early‐stage NLPHL do not receive radiation therapy at diagnosis. Given the rarity of this histologic subtype, we believe that it is unlikely that randomized trials will be conducted in this setting. As a result, we believe that large database analyses, such as the current one, can serve an important role in identifying disparities in care and improving patient outcomes.

## Conflict of Interest

The authors declare no disclaimers or conflict of interests.

## Supporting information


**Figure S1.** Overall survival following diagnosis among patients with early stage nodular lymphocyte predominant Hodgkin lymphoma in the National Cancer Database (2004–2012) comparing radiotherapy use to none.

**Figure S2.** Overall survival following diagnosis among patients with early stage nodular lymphocyte predominant Hodgkin lymphoma in the National Cancer Database (2004–2012) comparing radiotherapy use to chemotherapy use.

**Figure S3.** Overall survival following diagnosis among patients with early stage nodular lymphocyte predominant Hodgkin lymphoma in the National Cancer Database (2004–2012) comparing radiotherapy use to chemoradiotherapy use.

**Figure S4.** Overall survival following diagnosis among patients with early stage nodular lymphocyte predominant Hodgkin lymphoma in the National Cancer Database (2004–2012) comparing chemotherapy use to chemoradiotherapy use.

**Table S1.** Analysis of factors associated with time to death among patients with early stage nodular lymphocyte predominant Hodgkin lymphoma in the National Cancer Database 2004–2012 comparing radiotherapy use to none.
**Table S2.** Analysis of factors associated with time to death among patients with early stage nodular lymphocyte predominant Hodgkin lymphoma in the National Cancer Database 2004–2012 comparing radiotherapy use to chemotherapy use.
**Table S3.** Analysis of factors associated with time to death among patients with early stage nodular lymphocyte predominant Hodgkin lymphoma in the National Cancer Database 2004–2012 comparing radiotherapy use to chemoradiotherapy use.
