# Supplementary figures and images for: Adult nodular lymphocyte‐predominant Hodgkin lymphoma: treatment modality utilization and survival
Source: Cancer Med. 2018 Feb 26;7(4):1118–26. doi: 10.1002/cam4.1383 (PMC5911587; doi:10.1002/cam4.1383)

## Unadjusted Survival Curve

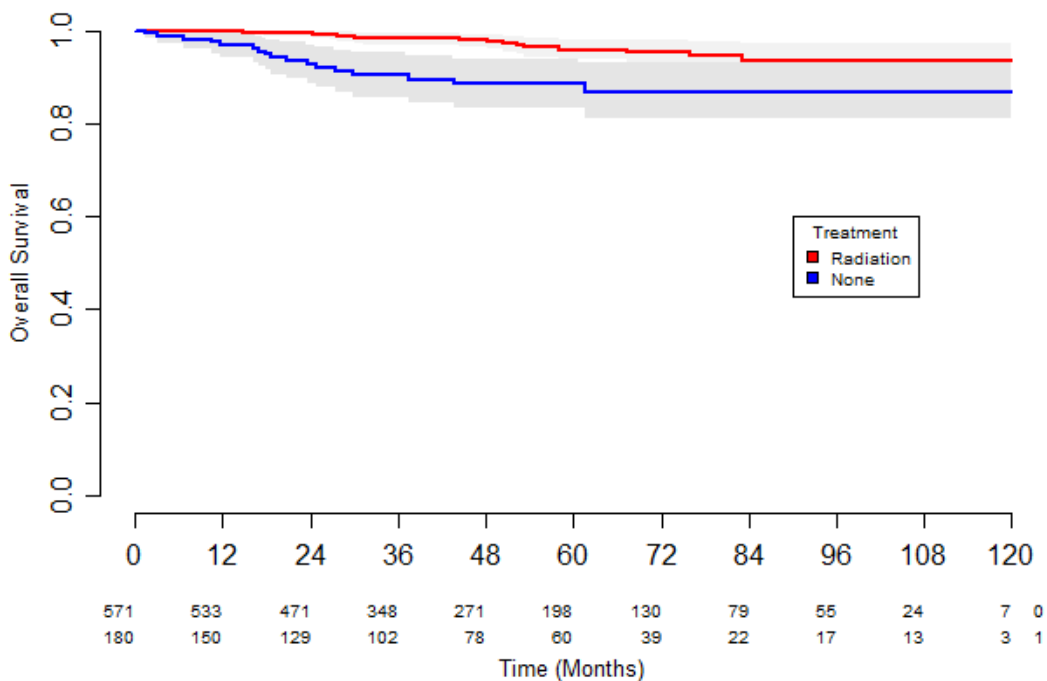

## Propensity Score Weighted Survival Curve

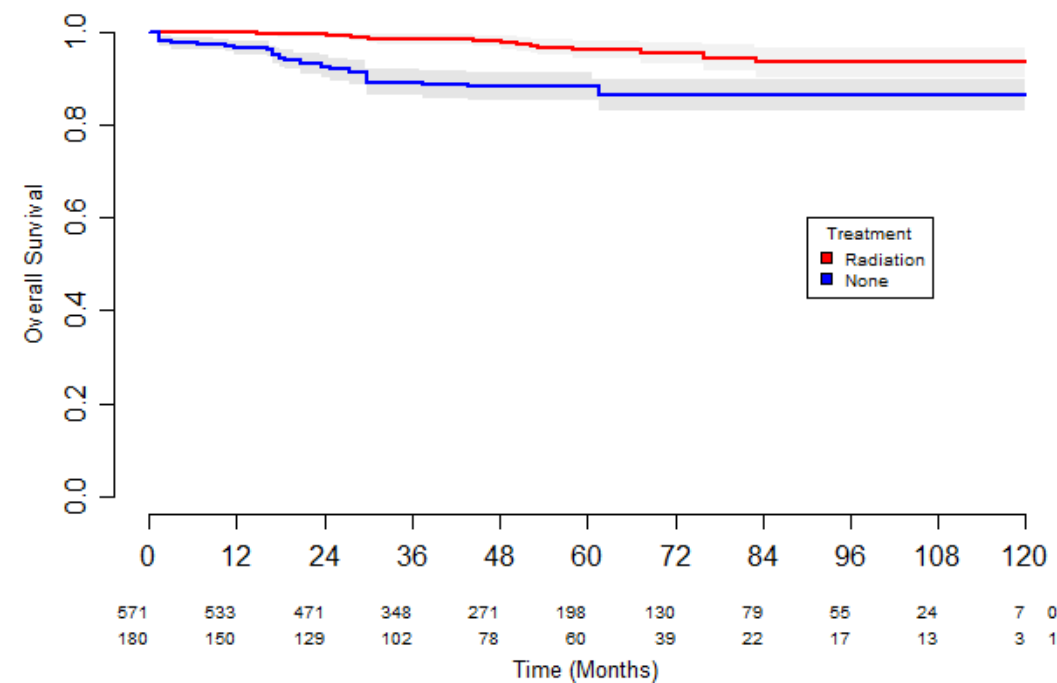

Supplement: Supplementary file 1 — Figure S1. Overall survival following diagnosis among patients with early stage nodular lymphocyte predominant Hodgkin lymphoma in the National Cancer Database (2004–2012) comparing radiotherapy use to none. [file CAM4-7-1118-s001.pdf]

## Unadjusted Survival Curve

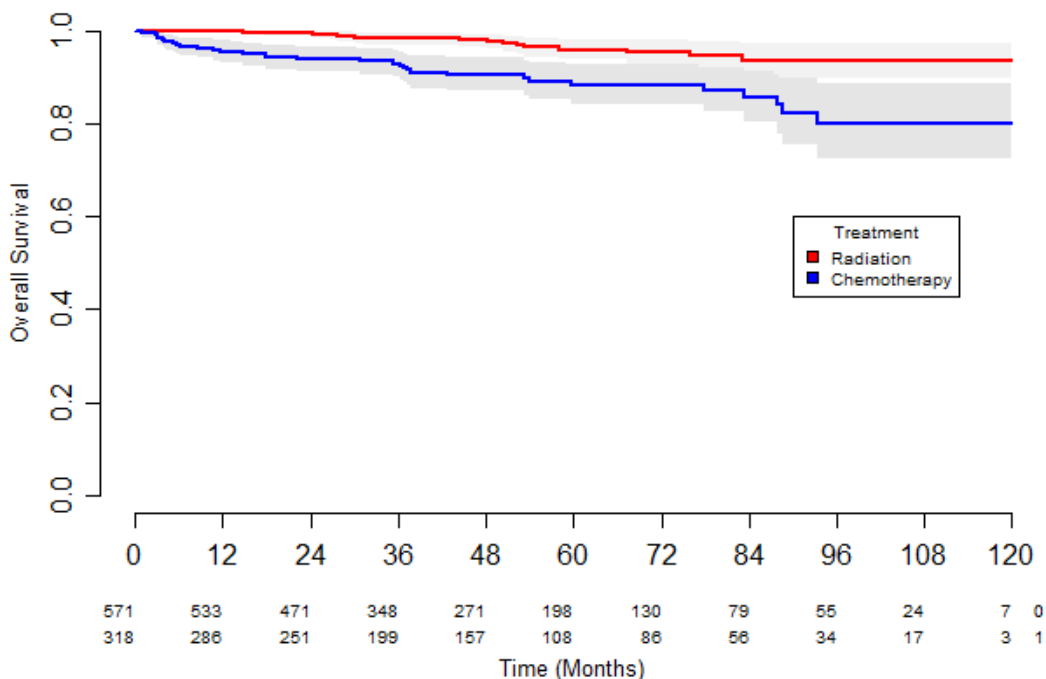

## Propensity Score Weighted Survival Curve

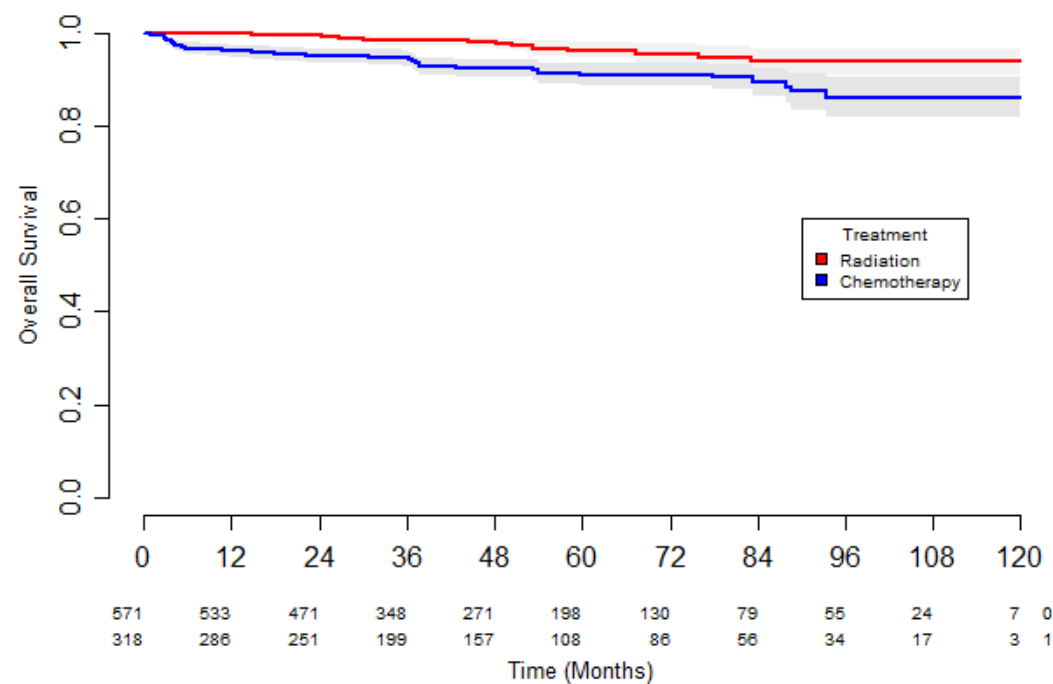

Supplement: Supplementary file 2 — Figure S2. Overall survival following diagnosis among patients with early stage nodular lymphocyte predominant Hodgkin lymphoma in the National Cancer Database (2004–2012) comparing radiotherapy use to chemotherapy use. [file CAM4-7-1118-s002.pdf]

## Unadjusted Survival Curve

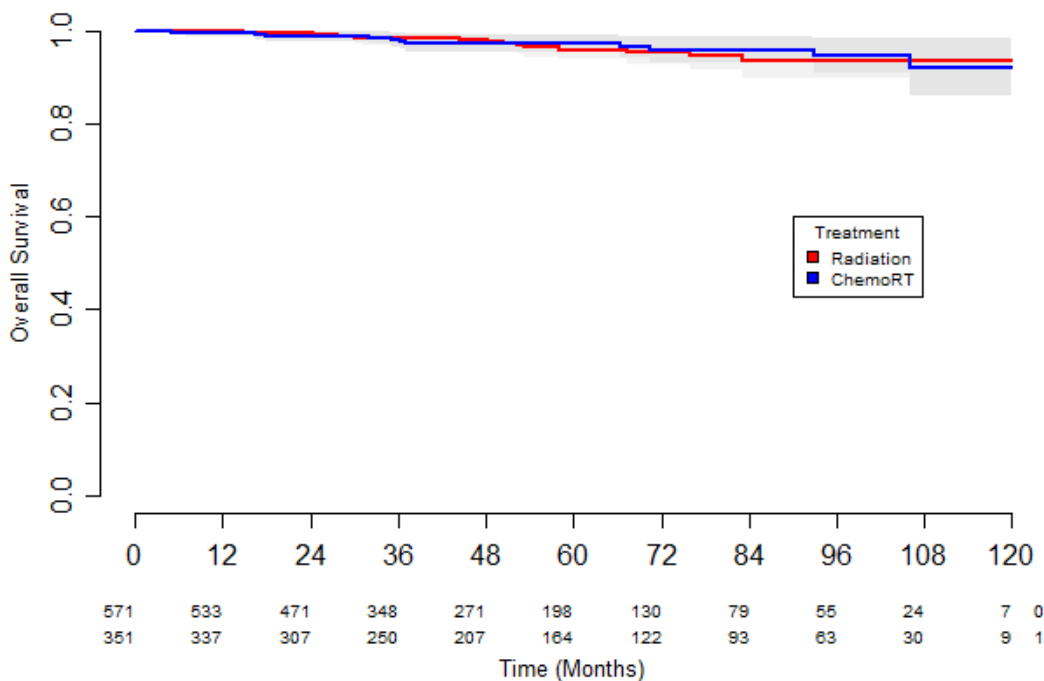

## Propensity Score Weighted Survival Curve

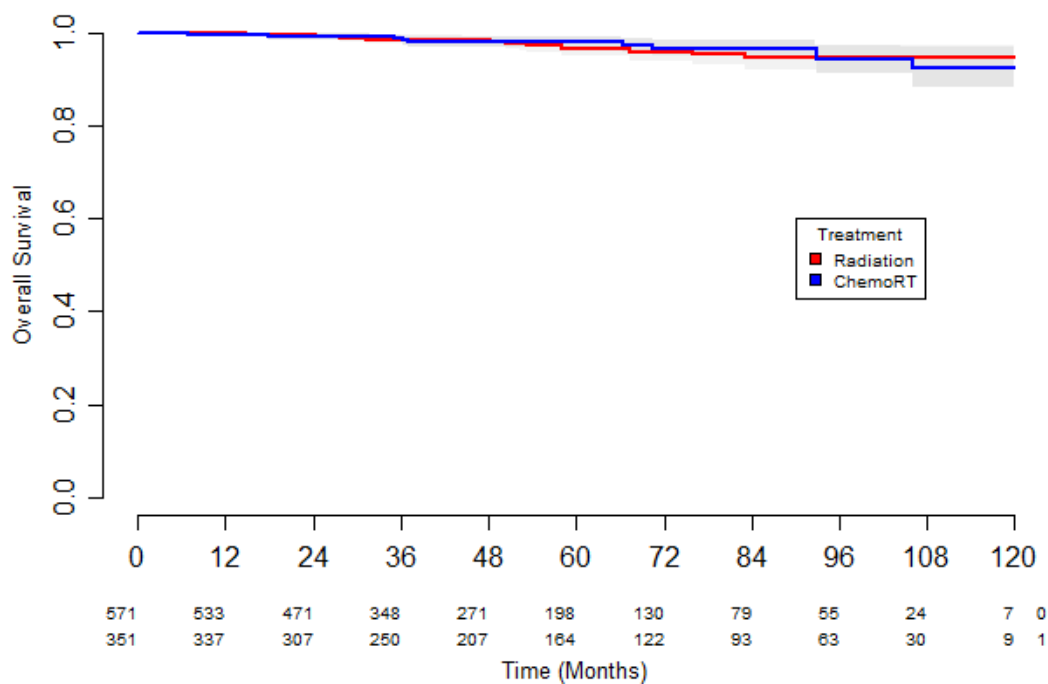

Supplement: Supplementary file 3 — Figure S3. Overall survival following diagnosis among patients with early stage nodular lymphocyte predominant Hodgkin lymphoma in the National Cancer Database (2004–2012) comparing radiotherapy use to chemoradiotherapy use. [file CAM4-7-1118-s003.pdf]

## Unadjusted Survival Curve

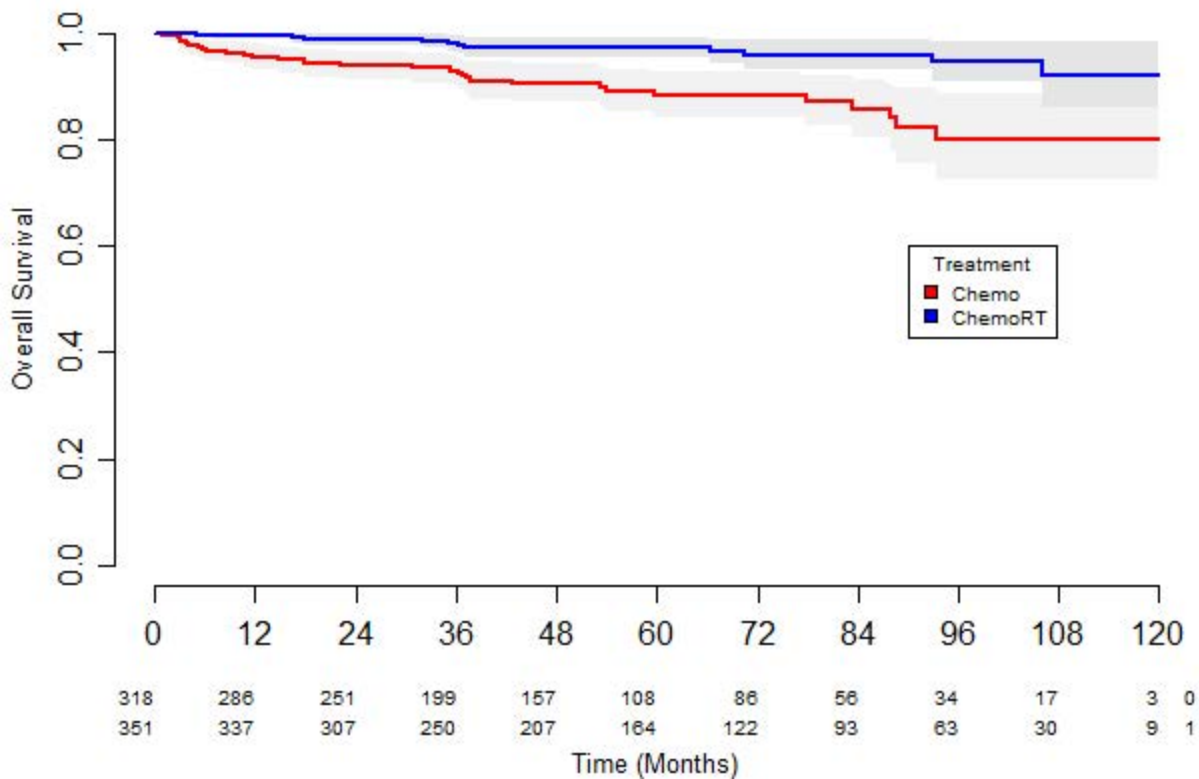

## Propensity Score Weighted Survival Curve

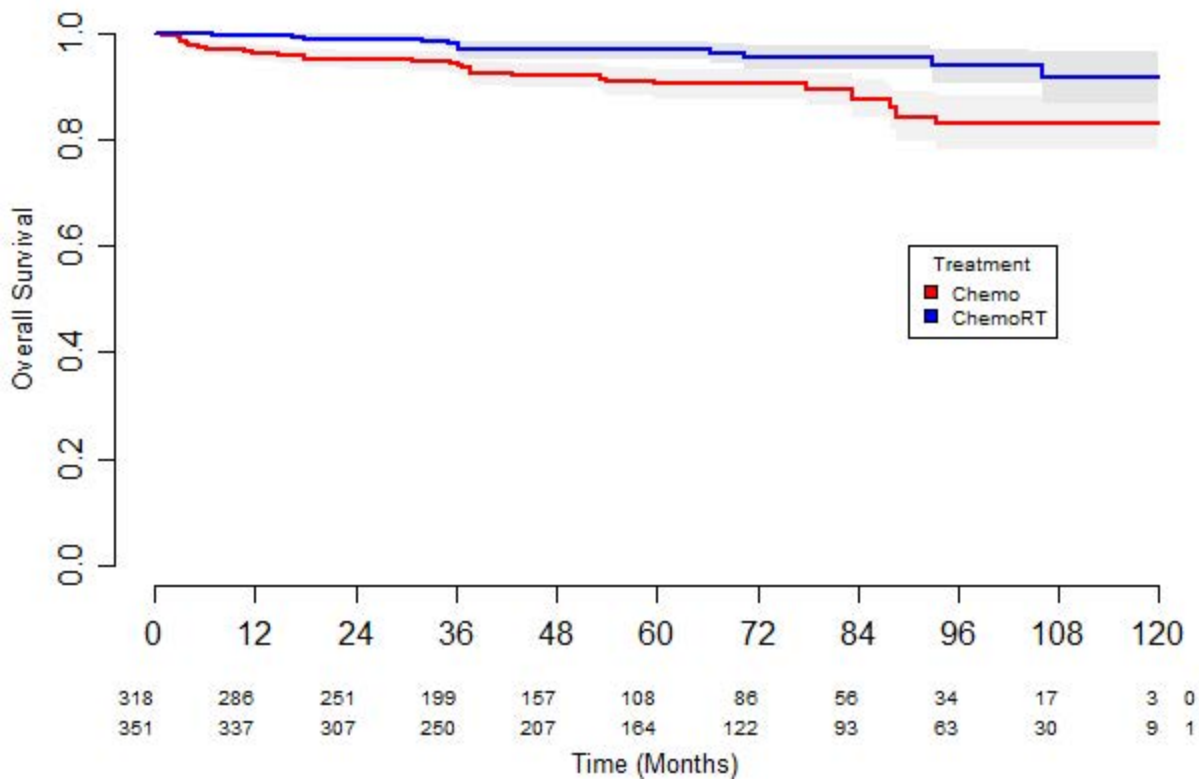

Supplement: Supplementary file 4 — Figure S4. Overall survival following diagnosis among patients with early stage nodular lymphocyte predominant Hodgkin lymphoma in the National Cancer Database (2004–2012) comparing chemotherapy use to chemoradiotherapy use. [file CAM4-7-1118-s004.pdf]
