# Supplementary material for: Adult nodular lymphocyte‐predominant Hodgkin lymphoma: treatment modality utilization and survival
Source: Cancer Med. 2018 Feb 26;7(4):1118–26. doi: 10.1002/cam4.1383 (PMC5911587; doi:10.1002/cam4.1383)
Supplement: Supplementary file 5 — Table S1. Analysis of factors associated with time to death among patients with early stage nodular lymphocyte predominant Hodgkin lymphoma in the National Cancer Database 2004–2012 comparing radiotherapy use to none. Table S2. Analysis of factors associated with time to death among patients with early stage nodular lymphocyte predominant Hodgkin lymphoma in the National Cancer Database 2004–2012 comparing radiotherapy use to chemotherapy use. Table S3. Analysis of factors associated with time to death among patients with early stage nodular lymphocyte predominant Hodgkin lymphoma in the National Cancer Database 2004–2012 comparing radiotherapy use to chemoradiotherapy use. [file CAM4-7-1118-s005.docx]

**SUPPLEMENTAL MATERIAL**

**Figure 1**: Overall survival following diagnosis among patients with early stage nodular lymphocyte predominant Hodgkin lymphoma in the National Cancer Database (2004-2012) comparing radiotherapy use to none.

**Figure 2**: Overall survival following diagnosis among patients with early stage nodular lymphocyte predominant Hodgkin lymphoma in the National Cancer Database (2004-2012) comparing radiotherapy use to chemotherapy use.

**Figure 3**: Overall survival following diagnosis among patients with early stage nodular lymphocyte predominant Hodgkin lymphoma in the National Cancer Database (2004-2012) comparing radiotherapy use to chemoradiotherapy use.

**Figure 4**: Overall survival following diagnosis among patients with early stage nodular lymphocyte predominant Hodgkin lymphoma in the National Cancer Database (2004-2012) comparing chemotherapy use to chemoradiotherapy use.

**Table 1**: Analysis of factors associated with time to death among patients with early stage nodular lymphocyte predominant Hodgkin lymphoma in the National Cancer Database 2004-2012 comparing radiotherapy use to none.

**Table 2**: Analysis of factors associated with time to death among patients with early stage nodular lymphocyte predominant Hodgkin lymphoma in the National Cancer Database 2004-2012 comparing radiotherapy use to chemotherapy use.

**Table 3**: Analysis of factors associated with time to death among patients with early stage nodular lymphocyte predominant Hodgkin lymphoma in the National Cancer Database 2004-2012 comparing radiotherapy use to chemoradiotherapy use.

**Table 1**: Analysis of factors associated with time to death among patients with early stage nodular lymphocyte predominant Hodgkin lymphoma in the National Cancer Database 2004-2012 comparing radiotherapy use to none.

|  |  | **Unadjusted** | | | **Multivariable Cox model** | | | **Propensity Score weighted** | | |
| --- | --- | --- | --- | --- | --- | --- | --- | --- | --- | --- |
|  |  | *HR* | *95% CI* | *p-value* | *HR* | *95% CI* | *p-value* | *HR* | *95% CI* | *p-value* |
| ***Therapy*** | |  |  |  |  |  |  |  |  |  |
| None | | 1.000 | - | - | 1.000 | - | - | 1.000 | - | - |
| Radiation | | 0.297 | 0.153-0.577 | <0.001 | 0.305 | 0.152-0.611 | 0.001 | 0.272 | 0.167-0.442 | <0.001 |
| ***Patient age*** | |  |  |  |  |  |  |  |  |  |
| <= 60 | |  |  |  | 1.000 | - | - |  |  |  |
| > 60 | |  |  |  | 4.531 | 2.227-9.22 | <0.001 |  |  |  |
| ***Year of Diagnosis*** | |  |  |  |  |  |  |  |  |  |
| 2004 | |  |  |  |  |  |  |  |  |  |
| 2005 | |  |  |  |  |  |  |  |  |  |
| 2006 | |  |  |  |  |  |  |  |  |  |
| 2007 | |  |  |  |  |  |  |  |  |  |
| 2008 | |  |  |  |  |  |  |  |  |  |
| 2009 | |  |  |  |  |  |  |  |  |  |
| 2010 | |  |  |  |  |  |  |  |  |  |
| 2011 | |  |  |  |  |  |  |  |  |  |
| 2012 | |  |  |  |  |  |  |  |  |  |
| ***Sex*** | |  |  |  |  |  |  |  |  |  |
| Male | |  |  |  |  |  |  |  |  |  |
| Female | |  |  |  |  |  |  |  |  |  |
| ***Race*** | |  |  |  |  |  |  |  |  |  |
| White | |  |  |  |  |  |  |  |  |  |
| Black | |  |  |  |  |  |  |  |  |  |
| American Indian | |  |  |  |  |  |  |  |  |  |
| Asian/Pacific Islander | |  |  |  |  |  |  |  |  |  |
| Unknown | |  |  |  |  |  |  |  |  |  |
| Hispanic | |  |  |  |  |  |  |  |  |  |
| ***Median income of zip*** | |  |  |  |  |  |  |  |  |  |
| <$38,000 | |  |  |  |  |  |  |  |  |  |
| $38,000-$47,999 | |  |  |  |  |  |  |  |  |  |
| $48,000-$62,999 | |  |  |  |  |  |  |  |  |  |
| $63,000 + | |  |  |  |  |  |  |  |  |  |
|  | Unknown |  |  |  |  |  |  |  |  |  |
| ***Distance to Hospital*** | |  |  |  |  |  |  |  |  |  |
| < 25 mi | |  |  |  | 1.000 | - | - |  |  |  |
| 25-100 mi | |  |  |  | 1.260 | 0.482-3.289 | 0.637 |  |  |  |
| > 100 mi | |  |  |  | 0.000 | NA | 0.996 |  |  |  |
| Unknown | |  |  |  | 2.117 | 0.275-16.326 | 0.472 |  |  |  |
| ***Charlson/Deyo Score*** | |  |  |  |  |  |  |  |  |  |
| 0 | |  |  |  | 1.000 | - | - |  |  |  |
| 1 | |  |  |  | 1.486 | 0.628-3.517 | 0.367 |  |  |  |
| 2 | |  |  |  | 6.080 | 1.7-21.745 | 0.006 |  |  |  |
| ***Insurance*** | |  |  |  |  |  |  |  |  |  |
| No | |  |  |  |  |  |  |  |  |  |
| Yes | |  |  |  |  |  |  |  |  |  |
| ***Stage*** | |  |  |  |  |  |  |  |  |  |
| Stage I | |  |  |  | 1.000 | - | - |  |  |  |
| Stage II | |  |  |  | 1.689 | 0.843-3.384 | 0.139 |  |  |  |

**Table 2**: Analysis of factors associated with time to death among patients with early stage nodular lymphocyte predominant Hodgkin lymphoma in the National Cancer Database 2004-2012 comparing radiotherapy use to chemotherapy use.

|  |  | **Unadjusted** | | | **Multivariable Cox model** | | | **Propensity Score weighted** | | |
| --- | --- | --- | --- | --- | --- | --- | --- | --- | --- | --- |
|  |  | *HR* | *95% CI* | *p-value* | *HR* | *95% CI* | *p-value* | *HR* | *95% CI* | *p-value* |
| ***Therapy*** | |  |  |  |  |  |  |  |  |  |
| Chemotherapy | | 1.000 | - | - | 1.000 | - | - | 1.000 | - | - |
| Radiation | | 0.285 | 0.159-0.511 | <0.001 | 0.391 | 0.212-0.720 | 0.003 | 0.359 | 0.224-0.575 | <0.001 |
| ***Patient age*** | |  |  |  |  |  |  |  |  |  |
| <= 60 | |  |  |  | 1.000 | - | - |  |  |  |
| > 60 | |  |  |  | 3.402 | 1.904-6.079 | <0.001 |  |  |  |
| ***Year of Diagnosis*** | |  |  |  |  |  |  |  |  |  |
| 2004 | |  |  |  |  |  |  |  |  |  |
| 2005 | |  |  |  |  |  |  |  |  |  |
| 2006 | |  |  |  |  |  |  |  |  |  |
| 2007 | |  |  |  |  |  |  |  |  |  |
| 2008 | |  |  |  |  |  |  |  |  |  |
| 2009 | |  |  |  |  |  |  |  |  |  |
| 2010 | |  |  |  |  |  |  |  |  |  |
| 2011 | |  |  |  |  |  |  |  |  |  |
| 2012 | |  |  |  |  |  |  |  |  |  |
| ***Sex*** | |  |  |  |  |  |  |  |  |  |
| Male | |  |  |  |  |  |  |  |  |  |
| Female | |  |  |  |  |  |  |  |  |  |
| ***Race*** | |  |  |  |  |  |  |  |  |  |
| White | |  |  |  |  |  |  |  |  |  |
| Black | |  |  |  |  |  |  |  |  |  |
| American Indian | |  |  |  |  |  |  |  |  |  |
| Asian/Pacific Islander | |  |  |  |  |  |  |  |  |  |
| Unknown | |  |  |  |  |  |  |  |  |  |
| Hispanic | |  |  |  |  |  |  |  |  |  |
| ***Median income of zip*** | |  |  |  |  |  |  |  |  |  |
| <$38,000 | |  |  |  |  |  |  |  |  |  |
| $38,000-$47,999 | |  |  |  |  |  |  |  |  |  |
| $48,000-$62,999 | |  |  |  |  |  |  |  |  |  |
| $63,000 + | |  |  |  |  |  |  |  |  |  |
|  | Unknown |  |  |  |  |  |  |  |  |  |
| ***Distance to Hospital*** | |  |  |  |  |  |  |  |  |  |
| < 25 mi | |  |  |  | 1.000 | - | - |  |  |  |
| 25-100 mi | |  |  |  | 0.742 | 0.287-1.914 | 0.537 |  |  |  |
| > 100 mi | |  |  |  | 1.248 | 0.297-5.24 | 0.762 |  |  |  |
| Unknown | |  |  |  | 5.733 | 1.986-16.555 | 0.001 |  |  |  |
| ***Charlson/Deyo Score*** | |  |  |  |  |  |  |  |  |  |
| 0 | |  |  |  | 1.000 | - | - |  |  |  |
| 1 | |  |  |  | 2.692 | 1.363-5.316 | 0.004 |  |  |  |
| 2 | |  |  |  | 4.029 | 1.346-12.056 | 0.013 |  |  |  |
| ***Insurance*** | |  |  |  |  |  |  |  |  |  |
| No | |  |  |  |  |  |  |  |  |  |
| Yes | |  |  |  |  |  |  |  |  |  |
| ***Stage*** | |  |  |  |  |  |  |  |  |  |
| Stage I | |  |  |  | 1.000 | - | - |  |  |  |
| Stage II | |  |  |  | 2.010 | 1.107-3.651 | 0.022 |  |  |  |

**Table 3**: Analysis of factors associated with time to death among patients with early stage nodular lymphocyte predominant Hodgkin lymphoma in the National Cancer Database 2004-2012 comparing radiotherapy use to chemoradiotherapy use.

|  |  | **Unadjusted** | | | **Multivariable Cox model** | | | **Propensity Score weighted** | | |
| --- | --- | --- | --- | --- | --- | --- | --- | --- | --- | --- |
|  |  | *HR* | *95% CI* | *p-value* | *HR* | *95% CI* | *p-value* | *HR* | *95% CI* | *p-value* |
| ***Therapy*** | |  |  |  |  |  |  |  |  |  |
| Radiation | | 1.000 | - | - | 1.000 | - | - | 1.000 | - | - |
| Both | | 0.843 | 0.404-1.761 | 0.649 | 1.063 | 0.489-2.311 | 0.877 | 0.831 | 0.468-1.478 | 0.529 |
| ***Patient age*** | |  |  |  |  |  |  |  |  |  |
| <= 60 | |  |  |  | 1.000 | - | - |  |  |  |
| > 60 | |  |  |  | 6.917 | 3.193-14.987 | <0.001 |  |  |  |
| ***Year of Diagnosis*** | |  |  |  |  |  |  |  |  |  |
| 2004 | |  |  |  |  |  |  |  |  |  |
| 2005 | |  |  |  |  |  |  |  |  |  |
| 2006 | |  |  |  |  |  |  |  |  |  |
| 2007 | |  |  |  |  |  |  |  |  |  |
| 2008 | |  |  |  |  |  |  |  |  |  |
| 2009 | |  |  |  |  |  |  |  |  |  |
| 2010 | |  |  |  |  |  |  |  |  |  |
| 2011 | |  |  |  |  |  |  |  |  |  |
| 2012 | |  |  |  |  |  |  |  |  |  |
| ***Sex*** | |  |  |  |  |  |  |  |  |  |
| Male | |  |  |  |  |  |  |  |  |  |
| Female | |  |  |  |  |  |  |  |  |  |
| ***Race*** | |  |  |  |  |  |  |  |  |  |
| White | |  |  |  |  |  |  |  |  |  |
| Black | |  |  |  |  |  |  |  |  |  |
| American Indian | |  |  |  |  |  |  |  |  |  |
| Asian/Pacific Islander | |  |  |  |  |  |  |  |  |  |
| Unknown | |  |  |  |  |  |  |  |  |  |
| Hispanic | |  |  |  |  |  |  |  |  |  |
| ***Median income of zip*** | |  |  |  |  |  |  |  |  |  |
| <$38,000 | |  |  |  |  |  |  |  |  |  |
| $38,000-$47,999 | |  |  |  |  |  |  |  |  |  |
| $48,000-$62,999 | |  |  |  |  |  |  |  |  |  |
| $63,000 + | |  |  |  |  |  |  |  |  |  |
|  | Unknown |  |  |  |  |  |  |  |  |  |
| ***Distance to Hospital*** | |  |  |  |  |  |  |  |  |  |
| < 25 mi | |  |  |  | 1.000 | - | - |  |  |  |
| 25-100 mi | |  |  |  | 1.684 | 0.678-4.18 | 0.261 |  |  |  |
| > 100 mi | |  |  |  | 0.000 | NA | 0.996 |  |  |  |
| Unknown | |  |  |  | 4.574 | 0.597-35.041 | 0.143 |  |  |  |
| ***Charlson/Deyo Score*** | |  |  |  |  |  |  |  |  |  |
| 0 | |  |  |  | 1.000 | - | - |  |  |  |
| 1 | |  |  |  | 2.782 | 1.155-6.702 | 0.023 |  |  |  |
| 2 | |  |  |  | 1.206 | 0.156-9.325 | 0.858 |  |  |  |
| ***Insurance*** | |  |  |  |  |  |  |  |  |  |
| No | |  |  |  |  |  |  |  |  |  |
| Yes | |  |  |  |  |  |  |  |  |  |
| ***Stage*** | |  |  |  |  |  |  |  |  |  |
| Stage I | |  |  |  | 1.000 | - | - |  |  |  |
| Stage II | |  |  |  | 1.306 | 0.617-2.763 | 0.485 |  |  |  |
